# The Rise and Fall of Hydroxychloroquine for the Treatment and Prevention of COVID-19

**DOI:** 10.4269/ajtmh.20-1320

**Published:** 2020-11-24

**Authors:** Zelyn Lee, Craig R. Rayner, Jamie I. Forrest, Jean B. Nachega, Esha Senchaudhuri, Edward J. Mills

**Affiliations:** 1 University of Toronto, Toronto, Canada;; 2 Certara Inc, Princeton, New Jersey;; 3 Monash Institute of Pharmaceutical Sciences, Monash University, Melbourne, Australia;; 4 Cytel Inc., Vancouver, Canada;; 5 School of Population and Public Health, Faculty of Medicine, University of British Columbia, Vancouver, Canada;; 6 Department of Medicine, Stellenbosch University, Cape Town, South Africa;; 7 Department of Epidemiology, Johns Hopkins Bloomberg School of Public Health, Baltimore, Maryland;; 8 Department of International Health, Johns Hopkins Bloomberg School of Public Health, Baltimore, Maryland;; 9 Department of Epidemiology, University of Pittsburgh Graduate School of Public Health, Pittsburgh, Pennsylvania;; 10 Department of Infectious Diseases and Microbiology, University of Pittsburgh Graduate School of Public Health, Pittsburgh, Pennsylvania;; 11 Department of Health Research Evidence and Impact, Faculty of Health Sciences, McMaster University, Hamilton, Canada

## Abstract

The efficacy and safety of hydroxychloroquine (HCQ) for the prevention and treatment of COVID-19 has received great attention, and most notably, the enthusiasm for HCQ has been one of politicization rather than science. Laboratory studies and case series published early in the pandemic supported its efficacy. The scientific community raced to conduct observational and randomized evaluations of the drug in all stages of the disease, including prophylaxis, early treatment, and advanced disease. Yet a divisive media response affected recruitment, funding, and subsequent enthusiasm for continuing scientific investigations. Of the more than 300 HCQ trials registered, fewer than 50% report having recruited any patients, and most trials might fail to achieve any useful portions of their intended sample size. Multiple observational studies and two large randomized trials have demonstrated HCQ does not offer efficacy against COVID-19 in hospitalized patients. Prophylaxis studies and early treatment studies provided heterogeneous results and are plagued by low event rates and poor study outcome monitoring. Emerging high-quality evaluations of prophylaxis and early treatment do not support a role for HCQ in these populations. The story of HCQ for COVID-19 has followed a pattern of initial enthusiasm supported by poor quality evidence, followed by disappointment based on more rigorous evaluations. The experience of HCQ in the COVID-19 era calls for the depoliticization of science away from media glare.

The SARS-CoV-2 (COVID-19) pandemic has ushered unprecedented global collaboration by clinical scientists to identify candidate therapeutics for the treatment of COVID-19. Efforts rapidly identified effective therapies against advanced COVID-19, including remdesivir,^1^ shown to reduce the duration of hospitalization, and dexamethasone,^2^ shown to reduce mortality among severely sick patients. But in this global effort to identify effective therapies against COVID-19, no drug has been scrutinized nor generated more scientific and political commentary, than hydroxychloroquine (HCQ). The aim of this commentary was to provide a narrative of the political and scientific story of HCQ in the COVID-19 pandemic era to understand how the drug became so contentious in the public, political, and scientific arenas.

In early 2020, as the COVID-19 pandemic spread, the urgency to identify an effective therapy became evident. Repurposing already existing medicines is a priority due to their known safety profiles, availability, and ease of administrative issues. Hydroxychloroquine was identified as an early potential therapeutic candidate, drawing on evidence from reports of both in vitro and in vivo testing. Hydroxychloroquine is an inexpensive and globally accessible drug listed on the WHO’s list of Essential Medicines, approved for the treatment of rheumatic diseases and as both a treatment and prophylaxis for malaria.^3^ In March 2020, a study published by Yao et al.^4^ reported findings from an in vitro study that supported a loading dose of 400 mg twice daily of HCQ sulfate given orally, followed by a maintenance dose of 200 mg given twice daily for 4 days to treat SARS-CoV-2 infection. These findings were then used as justification for further clinical evaluation of HCQ as a treatment for COVID-19.

The first clinical report evaluating HCQ for the treatment of COVID-19 was an observational study of 20 patients treated in France with HCQ and found at day 6 post-inclusion, 70% of HCQ-treated patients were virologically cured compared with 12.5% in a control group (reported as highly significant, *P* = 0.001). The investigators reported that HCQ showed dramatic clinical benefit in reducing viral load and disease symptoms.^5^ Many methodological issues were noted about these initial reports, including small sample sizes, lack of randomization, lack of inclusion criteria, and overinterpretation of findings.^6,7^ These study findings were then widely reported in the medical press and by political leaders, resulting in the issuance of an emergency use authorization for HCQ in the United States (US), France, Ukraine, and Turkey, among others.^8^

This media attention resulted in a remarkable rise in both the off-label use of HCQ and interest within the clinical research community. The number of clinical trial registrations that aimed to assess the efficacy and safety of HCQ (www.covid19-trials.com) increased dramatically. At the same time, evidence emerged that COVID-19 is not a single disease but represents a spectrum of disease, from prophylaxis populations, to early treatment and hospitalized patients who may eventually require intensive care. The clinical research world was inadequately aware of the disease stages and so a plethora of trials evaluating antivirals in severe disease as well as application of findings from hospitalized patients to ambulatory patients. As of October 30, 2020, there were 2,462 trials registered to evaluate interventions for COVID-19; 341 of these trials intended to evaluate HCQ. Trial registrations for evaluations of HCQ peaked in April 2020, where more than half of registered trials intended to recruit patients in the hospitalized setting (Figure 1). Although registration of a trial indicates an intention to conduct study, it does not however necessarily confirm whether a registered trial was ever initiated.

**Figure 1.**
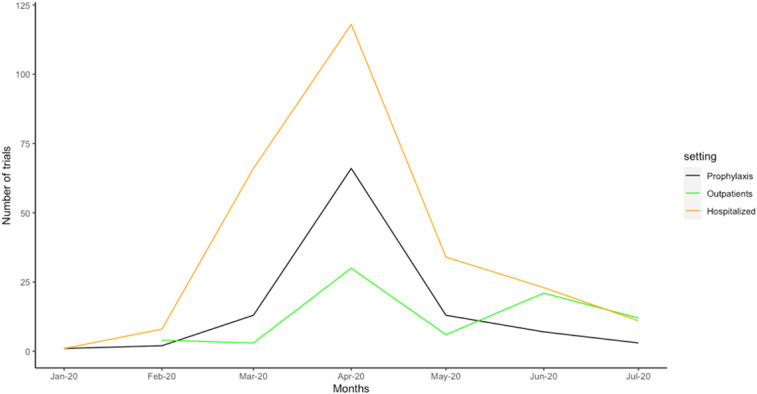
Number of active registered trials investigating hydroxychloroquine by date among patient populations of prophylaxis, outpatient, and hospitalized settings. This figure appears in color at www.ajtmh.org.

As trial registrations grew, observational study reports also emerged. These findings were generated nearly exclusively from studies among hospitalized populations, often in single hospital settings, and reported widely heterogeneous results.^9–11^ Some of these studies demonstrated a clinical benefit for the use of HCQ/CQ, whereas others showed no difference to placebo or standard of care.^12^ These observational studies also raised concern for cardiotoxic harm associated with the use of HCQ.^13^ By May 2020, as published results among hospitalized patients from predominantly Asian countries began to appear, it became clear that the evidence for HCQ as a treatment of COVID-19 was inconsistent and contradictory.^14^ Among the first 12 reports evaluating the efficacy of HCQ using both observational studies and clinical trials, in a combined sample of 3,543 hospitalized patients, there was no clear evidence to support continued use of this drug for hospitalized patient populations.^15^

The narrative on HCQ/CQ importantly shifted in May 2020 when the *Lancet* published and then retracted a study reporting no benefits of HCQ/CQ, after revelations surfaced that the authors’ study conclusions were based on unsubstantiated data.^16^ The study alleged to report on 96,032 patients across 671 hospitals worldwide and showed an increased risk of de-novo ventricular arrhythmia with treatment with HCQ. Although retracted, the messaging about associated risk of cardiac events, specifically extended QT intervals, led researchers, clinicians, and trial funders to become more skeptical of the safety and toxicity of HCQ.^17^ This had a dramatic effect on the number of new HCQ trials registrations (Figure 1) from April 2020 onward and resulted in trial funding abruptly ending and study ethics approvals rescinded for some of the largest planned HCQ trials.^18^

In July 2020, in the UK, the Randomised Evaluation of COVID-19 therapy (RECOVERY) trial reported results that showed potential harm with the use of HCQ in the treatment of COVID-19 among hospitalized patients (risk ratio for death at 28 days, 0.92; 95% CI: 0.85–0.99).^19^ This report further dissolved much remaining enthusiasm for therapeutic effects of HCQ in the clinical trial community evaluating hospitalized patients. Funding for HCQ trials also became increasingly more difficult to secure. The WHO Solidarity trial, for example, a multinational adaptive platform trial among hospitalized patients with COVID-19, was temporarily placed on hold, as was the COPCOV trial that intended to enroll forty thousand health workers to evaluate HCQ as prophylaxis.^20^ Subsequently, the CROWN Coronation Trial, evaluating HCQ as a prophylactic among 30,000 health workers, switched the HCQ arm to a measles, mumps, and rubella vaccine.^21^

As one might expect, the number of registered trials and the number of trials recruiting showed important differences. In the first two quarters of the year, 241 HCQ or CQ trials were registered. By July 1, 2020, only 51% of trials had begun recruitment. This decline in clinical trial registrations for HCQ evaluation continued into the months of July and August 2020. Clinical trial registrations in hospitalized populations dropped most substantially, but so did those in prophylaxis and outpatient settings (Figure 1).

There are several reasons a trial may not recruit or may only recruit a small proportion of the intended population. Chief among these reasons are insufficient funding and an insufficient supply of patients. Yet it is important to recognize that a trial that failed to recruit its intended sample size should not be considered a failure but rather a reason for possible collaboration. For example, the vast majority of clinical trials among early treatment populations in the United States were unable to recruit because of testing delays, and so a trial that intends to recruit, for example, 500 patients may only ever hypothetically recruit 50 patients. However, collaboration between 10 such trials will reach the target sample size. There are now several initiatives to promote collaboration and sharing of data. These include the Gates Medical Research Institute (GMRI), the UK Workbench, and the NIH Active Collaboration. Specifically, the GMRI has reached out to trialists evaluating HCQ in early treatment populations to formally commit to sharing data regardless of the sample size recruited, with the intention of increasing statistical power through each additional collaboration. This is conceptually an individual patient data meta-analysis and is likely the only strategy that will yield findings for HCQ.

Scientific consensus has now emerged that there is a lack of clinical benefit for using HCQ/CQ among hospitalized patients. Both the UK RECOVERY and the WHO SOLIDARITY trials (rate ratio: 1.19; 95% CI: 0.89–1.59) have now reported their findings on HCQ among hospitalized patients and failed to detect any benefit.^22^ On June 25, 2020, the U.S. The Food and Drug Administration announced it had revoked the emergency use authorization for HCQ and CQ.^23^ But, while enthusiasm for HCQ to treat COVID-19 has dwindled, there was a rational argument for continuing investigations of HCQ as prophylaxis or its effect in the early phase of the disease.^24^ Hydroxychloroquine has antiviral activity against SARS-CoV-2 in laboratory studies through a number of direct molecular actions and anti-inflammatory and immunomodulatory effects,^25^ and respected voices argued that if HCQ were to play an important role, it would be in prophylaxis or early treatment of the disease, when it was primarily a viral infection stage of the disease.^26^ Several pre-exposure and postexposure prophylaxis trials have now been reported. A meta-analysis evaluating HCQ prophylaxis (both pre- and postexposure) reported a significant pooled risk reduction of 22% (95% CI: 1–39) based on four trials.^27^ Although this generated enthusiasm again that HCQ may have a role in this pandemic, updating this with two new randomized controlled trials (RCTs) of prophylaxis with nonsignificant findings changes the detected effect importantly (risk reduction 16%, 95% CI: −2 to 30).^28^

Similarly, early treatment of COVID-19 with HCQ initially yielded disparate results. Among 10 RCTs enrolling 2,535 patients, outcomes reporting on viral clearance after an average of 7 days of treatment were largely negative for HCQ treatment, and the number of hospitalizations reported in the trials was only 3.6%, indicating that detecting whether HCQ prevents disease progression in clinical trials is statistically difficult.^29^ Early treatment trials in COVID-19 are particularly challenging as we are aware that most patients, regardless of risk profiles, will clear the virus irrespective of treatment, and only a small number will progress to hospitalization and death. In the most detailed of the early treatment trials with regard to viral monitoring. This study was terminated early for futility of clinical endpoints. The largest RCT using hospitalization as an outcome, from Brazil, found no effect of HCQ on hospitalizations or death and was similarly terminated because of futility (risk ratio: 1.00; 95% CI: 0.45–2.21, unpublished).

The story of HCQ clinical trials has been influenced strongly, by political endorsements, media scrutiny with a political agenda, and a lack of rigorous scientific debate.^31^ Widespread anxieties have been fueled by results being retracted in a scientific arena where information has become overtly politicized. Although we hope medicine can be apolitical, the trajectory of media interest in HCQ and its promotion by politicians has dramatically affected the funding, conduct, and interpretation of clinical trials.^17^ It is almost always the case that research priorities in medicine are determined through a combination of financial, political, and social factors; what is distinctive in this case is that both research priorities and research findings have been swayed by these elements.

Emerging evidence suggests that the scientific and political story of HCQ has been one of a rise and fall—initial enthusiasm and scientific signals supported by low-quality evidence, followed by subsequent disappointment and lack of statistical effects as more rigorous evaluations are reported. The story of HCQ and COVID-19 is an important one to document. There is a clear need for clinical trials to explore promising, scalable, and cost-efficient therapeutics and vaccines for COVID-19 globally, and an urgent need to allow sound science to guide public health policy, rather than politics or poor-quality designs.
